# Bioactive Constituents, Mechanisms, and Complementary Therapeutic Applications of Food–Medicine Continuum Materia Medica for Atherosclerosis Prevention and Treatment

**DOI:** 10.3390/ph19060856

**Published:** 2026-05-29

**Authors:** Xiaorong Zhang, Mengyue Dong, Xinke Wang, Yingjie Hong, Xin Zhang, Yonghuan Niu, Xuefeng Li

**Affiliations:** School of Basic Medical Sciences, Lanzhou University, Lanzhou 730000, China; zhxiaorong2025@lzu.edu.cn (X.Z.); dongmy2024@lzu.edu.cn (M.D.); wangxk2025@lzu.edu.cn (X.W.); hongyj2024@lzu.edu.cn (Y.H.); zhx2023@lzu.edu.cn (X.Z.); nyonghuan2023@lzu.edu.cn (Y.N.)

**Keywords:** food and medicine continuum, atherosclerosis, bioactive components, cardiovascular protection, functional foods

## Abstract

Cardiovascular disease (CVD) represents the leading cause of mortality worldwide, with atherosclerosis (AS) serving as its primary pathological foundation, involving multiple pathological processes, including lipid metabolism disorders, chronic inflammation, and endothelial dysfunction. The food and medicine continuum (FMC) concept originates from traditional Chinese medicine, emphasizing that certain foods possess both nutritional and medicinal value, aligning closely with the modern “food is medicine” philosophy. This narrative review examines the bioactive components and anti-atherosclerotic mechanisms of ten FMC materia medica: hawthorn fruit (*Crataegus* Fructus), ginkgo seed (*Ginkgo* Semen), milkvetch root (*Astragali* Radix), turmeric (*Curcumae longae* Rhizoma), ginger (*Zingiberis* Rhizoma Recens), glossy ganoderma (*Ganoderma*), Angelica sinensis (*Angelicae sinensis* Radix), barbary wolfberry fruit (*Lycii* Fructus), lotus leaf (*Nelumbinis* Folium), and honey (*Mel*). These materia medica are rich in bioactive constituents, including flavonoids, terpenoids, and polysaccharides, which can exert cardiovascular protective effects, such as regulating lipid metabolism, inhibiting inflammation and oxidative stress, improving endothelial function, and modulating gut microbiota. Regarding clinical evidence, meta-analyses support the beneficial effects of ginger and honey on cardiometabolic risk factors, though the field still faces challenges, including the need for higher-level clinical evidence and difficulties in product standardization. This review aims to integrate traditional knowledge with modern scientific approaches, providing scientific evidence for the development of functional foods and phytotherapy.

## 1. Introduction

Cardiovascular disease (CVD) has become a significant health threat worldwide. According to the 2023 Global Burden of Disease Study, CVD was responsible for 19.2 million deaths, making it the leading cause of mortality globally [1]. Atherosclerosis (AS) is the main pathological component of CVD and involves several complex biological processes [2]. Despite advancements in pharmacological therapies—including statins, ezetimibe, proprotein convertase subtilisin/kexin type 9 (PCSK9) monoclonal antibodies (evolocumab, alirocumab), the small interfering RNA agent inclisiran, and bempedoic acid—challenges such as residual cardiovascular risk, adverse drug reactions, medication non-adherence, and rising medical costs remain prominent. PCSK9 inhibitors reduce LDL-cholesterol by approximately 50–65% and significantly decrease major adverse cardiovascular events when added to maximally tolerated statin therapy [3,4,5]. Nevertheless, single-target treatments often fail to effectively address the multifaceted nature of AS pathology [6,7].

Recently, the role of functional foods in AS prevention has garnered widespread attention. Epidemiological studies demonstrate that plant-based dietary patterns are closely associated with a reduced risk of AS [8,9]. The “food is medicine” concept is embodied in multiple traditional medical systems globally, including the traditional Chinese medicine (TCM) theory of the “food and medicine continuum” (FMC), Mediterranean dietary traditions, and Indian Ayurvedic medicine [10,11]. The FMC emphasizes that many foods possess both nutritional and medicinal value, aligning closely with core concepts of modern nutrition and integrative medicine [12,13,14].

**Figure 1:**
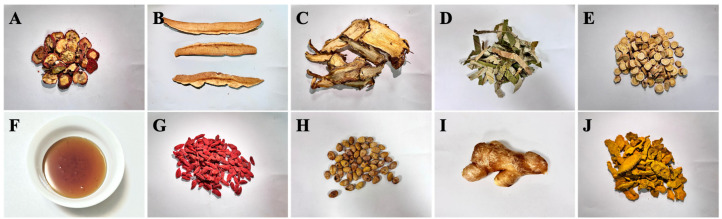
Ten food and medicine continuum decoction pieces. Note: (**A**) hawthorn fruit (*Crataegus* Fructus); (**B**) glossy ganoderma (*Ganoderma*); (**C**) Angelica sinensis (*Angelicae sinensis* Radix); (**D**) lotus leaf (*Nelumbinis* Folium); (**E**) milkvetch root (*Astragali* Radix); (**F**): honey (*Mel*); (**G**): barbary wolfberry fruit (*Lycii* Fructus); (**H**): fried ginkgo seed (*Ginkgo* Semen); (**I**): fresh ginger (*Zingiberis* Rhizoma Recens); (**J**): turmeric *(Curcumae longae* Rhizoma).

Bioactive components found in plant-derived foods serve as the foundational elements for their effects against atherosclerosis (AS). Key classes of these compounds include flavonoids, terpenoids, phenolic acids, alkaloids, and polysaccharides. They exert protective effects through various pathways, such as regulating lipid metabolism, inhibiting inflammation and oxidative stress, improving endothelial function, and modulating gut microbiota [15,16,17,18,19,20,21]. The combined use of modern analytical techniques, network pharmacology, and molecular docking technologies offers effective methods for identifying bioactive components and understanding their mechanisms of action in the FMC [22,23,24]. Clinical research provides significant evidence supporting the use of functional foods in the prevention and treatment of AS. Authoritative guidelines highlight that plant-based dietary patterns are fundamental to primary prevention of AS [9]. Nevertheless, there are challenges in translating laboratory research to clinical applications. These challenges include issues with low bioavailability, a lack of standardized extraction processes, and incomplete quality control systems. This article selects ten representative FMC materia medica from the 106 Chinese materia medica listed in China’s National Health Commission “Catalogue of Substances that are Both Food and Traditional Chinese Medicine”: hawthorn fruit (*Crataegus* Fructus), ginkgo seed (*Ginkgo* Semen), milkvetch root (*Astragali* Radix), turmeric (*Curcumae longae* Rhizoma), ginger (*Zingiberis* Rhizoma Recens), glossy ganoderma (*Ganoderma*), Angelica sinensis (*Angelicae sinensis* Radix), barbary wolfberry fruit (*Lycii* Fructus), lotus leaf (*Nelumbinis* Folium), and honey (*Mel*) (Figure 1). In the context of traditional Chinese medicine, the term “materia medica” encompasses both botanical and non-botanical substances, including animal-derived and mineral products.

Selection criteria included (1) official inclusion in China’s national FMC catalogue, confirming regulatory recognition of dual food–medicine status; (2) documented traditional use for cardiovascular protection in classical Chinese medical texts; and (3) availability of published preclinical research elucidating bioactive components and potential anti-atherosclerotic mechanisms. Other cardiovascular-relevant FMC catalogue items include American ginseng, which is a well-studied traditional FMC herb known for its protective effects on the cardiovascular system. However, there are currently no experimental studies investigating its effects on atherosclerosis. This review aims to examine the bioactive components, molecular mechanisms, and complementary applications of these materia medica for AS prevention. The goal is to provide scientific evidences for the development of functional foods and nutritional strategies for the prevention of AS.

## 2. Methodology

A comprehensive literature search was performed across the PubMed, Web of Science, Scopus, and China National Knowledge Infrastructure (CNKI) databases from inception to March 2026. The search strategy included (1) individual herb names, both Latin binomial and common names, combined with terms such as “atherosclerosis,” “cardiovascular,” “lipid metabolism,” “endothelial function,” or “inflammation”; and (2) “medicine–food homology” or “food–medicine continuum” combined with “bioactive compounds” or “cardiovascular protection.” Systematic reviews, meta-analyses, and randomized controlled trials were prioritized, followed by mechanistic studies in animal models and cell-based assays. Reference lists of retrieved articles and relevant reviews were manually screened to identify additional studies. All references were managed using Endnote software (Endnote 2025.1).

## 3. Bioactive Components of Food and Medicine Continuum Materia Medica

### 3.1. Flavonoid Compounds

Flavonoid compounds represent the most widely distributed class of bioactive components in FMC materia medica. Hawthorn fruit flavonoids are exemplified by hyperoside, quercetin, and procyanidin B2, with a total flavonoid content of 2–4%, regulating lipid metabolism through activation of adenosine 5′-monophosphate-activated protein kinase (AMPK)/sterol regulatory element-binding protein-1c (SREBP-1c) and peroxisome proliferator-activated receptor alpha (PPARα) pathways in cell-based and animal models [25,26,27,28]. Ginkgo seed contains flavonoid glycosides, primarily glycoside forms of quercetin, kaempferol, and isorhamnetin, along with unique biflavonoid compounds (ginkgetin, isoginkgetin, etc.) possessing antioxidant and anti-inflammatory activities [29,30,31,32]. Milkvetch root isoflavone components (calycosin, formononetin) exert cardioprotective effects through regulating ESR1 expression [33,34,35]. Lotus leaf and honey are also rich in flavonol compounds such as quercetin and kaempferol, exhibiting antioxidant and endothelial function-improving effects [36,37,38].

### 3.2. Terpenoid Compounds

Terpenoid compounds constitute important bioactive components responsible for the cardiovascular protective effects of FMC materia medica. Ginkgo seed terpene lactones represent unique bioactive constituents, including ginkgolides A, B, C, and bilobalide [39,40]. Ginkgolides are specific antagonists of platelet-activating factor (PAF), inhibiting PAF-induced platelet aggregation [41,42]; bilobalide exerts neuroprotective and antioxidant effects through activating the nuclear factor erythroid 2-related factor 2 (Nrf2) pathway [43].

In preclinical studies, milkvetch root saponin components are exemplified by astragaloside IV, with content ranging from 0.02 to 0.04%, improving vascular endothelial function through activating the phosphatidylinositol 3-kinase (PI3K)/protein kinase B (Akt) signaling pathway, increasing endothelial nitric oxide synthase (eNOS) phosphorylation, and promoting nitric oxide (NO) production [44,45,46,47,48,49]. Glossy ganoderma triterpenoid compounds belong to lanostane-type tetracyclic triterpenes, exemplified by ganoderic acid A, with content of approximately 1–3%, reducing cholesterol synthesis through inhibiting 3-hydroxy-3-methylglutaryl-coenzyme A (HMG-CoA) reductase activity and reducing inflammatory factor production through inhibiting nuclear factor kappa B (NF-κB) and mitogen-activated protein kinase (MAPK) signaling pathways in vitro [50,51]. Hawthorn fruit triterpenoid components (ursolic acid, oleanolic acid) upregulate antioxidant enzyme expression, including heme oxygenase-1 (HO-1) and NAD(P)H quinone oxidoreductase 1 (NQO1), through activating the Nrf2 pathway [52,53,54].

### 3.3. Polysaccharide Compounds

Polysaccharide compounds are significant bioactive components found in various FMC materia medica, known for their immunomodulatory and cardiovascular protective effects. Astragalus polysaccharide, with a molecular weight range of 10–1000 kDa and a content range of 10–20%, exhibits immunomodulatory effects and protects vascular endothelial cells. It activates macrophages and helps regulate the balance of T cell subsets [55,56,57,58]. Glossy ganoderma polysaccharides are characterized primarily by β-glucans and offer numerous cardiovascular protective benefits. These include immunomodulation, anti-inflammatory properties, antioxidative effects, and the regulation of glucose–lipid metabolism. Additionally, they help modulate gut microbiota composition and promote the production of short-chain fatty acids [59,60,61,62,63].

Barbary wolfberry fruit polysaccharides are arabinogalactan-type polysaccharides with a content ranging from 5 to 8%. They exhibit several cardiovascular protective effects, including antioxidant, anti-inflammatory, and immunomodulatory properties, and protection of mitochondrial function [64,65]. On the other hand, lotus leaf polysaccharides are rhamnogalacturonan-I (RG-I)-type pectins that effectively bind bile acids in the intestinal lumen with a binding rate of 60% to 80%, disrupting the enterohepatic circulation and promoting hepatic conversion of cholesterol to bile acids [66,67]. Mechanistically, pectin-type polysaccharides interact with bile acids through both direct molecular binding with bile salt monomers (via hydrogen bonds and van der Waals interactions mediated by galactan side chains) and viscosity-dependent physical entrapment of bile-salt-mixed micelles within the gel network [67,68]. In vitro studies on dietary fiber generally demonstrate greater adsorption of dihydroxy bile acids than trihydroxy bile acids, likely due to the greater hydrophobicity of dihydroxy species; however, specific affinity data for lotus leaf RG-I pectin toward individual bile acid species have not yet been reported [69,70]. They also inhibit cholesterol micelle solubility at an inhibition rate of approximately 50% and stimulate probiotic growth. These polysaccharides demonstrate greater effectiveness in regulating glucose and lipid metabolism compared to flavonoids and alkaloids [66,71].

### 3.4. Phenolic Acid and Gingerol Compounds

Phenolic acid compounds are commonly found in various materia medica used in traditional medicine. For instance, in *Angelica sinensis*, ferulic acid is a notable phenolic acid component, with a content of approximately 0.05–0.1%. This compound exhibits antithrombotic and vasodilatory properties by reducing platelet aggregation through the inhibition of platelet cyclooxygenase (COX) and thromboxane A2 (TXA2) synthesis. Additionally, it enhances vasodilation by increasing the production of nitric oxide (NO) from endothelial cells [72,73]. However, direct comparative data on the vasodilatory potency of ferulic acid relative to NO donors or endogenous NO are currently lacking, and the concentrations used in these in vitro and ex vivo studies (10^−5^ to 10^−3^ mol/L) may exceed physiologically achievable levels from dietary intake. Another important component in *Angelica sinensis* is ligustilide, a phthalide that constitutes around 45–55% of the total volatile oil. Ligustilide also demonstrates vasodilatory effects and helps prevent platelet aggregation [74,75]. Furthermore, chlorogenic acid, found in hawthorn fruit, is known for its antioxidant properties and its role in regulating glucose and lipid metabolism [76].

The primary bioactive component of turmeric is curcumin, which contains about 2–5% of this compound. It is known for its significant antioxidant and anti-inflammatory properties, effectively scavenging free radicals and inhibiting the NF-κB signaling pathway [77,78,79]. However, curcumin has extremely low oral bioavailability, typically less than 1%. This limitation is primarily due to poor water solubility, rapid metabolism in the intestines, and hepatic reductive metabolism [80,81]. Interestingly, ar-turmerone, a component found in turmeric’s volatile oil, can enhance curcumin’s bioavailability by approximately two to three times [82].

Ginger contains pungent compounds that are its primary bioactive components, with 6-gingerol being the most abundant, comprising approximately 25–35% of total gingerols [83,84,85]. 6-Gingerol has demonstrated antioxidant, anti-inflammatory, and antiplatelet aggregation properties, primarily by inhibiting the expression of COX-2 and inducible nitric oxide synthase (iNOS) [86,87]. Shogaols are dehydration products of gingerols formed during the drying or heating process and serve as the major bioactive components of dried ginger [88]. In vitro comparisons indicate that the antioxidant and anti-inflammatory activities of 6-shogaol are approximately two to three times greater than those of 6-gingerol. This enhanced activity is attributed to its α,β-unsaturated ketone structure, which activates the Nrf2/antioxidant response element (ARE) antioxidant pathway [89,90].

Honey contains a variety of phenolic acids, such as caffeic acid, ferulic acid, and chlorogenic acid, as well as flavonoid compounds like quercetin, kaempferol, and apigenin. The composition and content of these compounds can vary depending on the floral source [91]. Dark honeys, such as buckwheat honey and honeydew honey, typically have a higher concentration of phenolic compounds and exhibit greater antioxidant activity compared to lighter-colored honeys [38]. The phenolic compounds, enzymes, and organic acids found in honey work together to neutralize free radicals, regulate antioxidant enzyme activity, and reduce oxidative stress [92].

### 3.5. Other Bioactive Components

Lotus leaf alkaloid components are exemplified by nuciferine, accounting for approximately 50–70% of total alkaloids. It helps regulate lipids and reduce weight by activating the AMPK signaling pathway, which promotes fatty acid β-oxidation, inhibits fat production, and improves insulin resistance [93]. Neferine, another alkaloid, has antiarrhythmic and antihypertensive effects. It acts like a class III antiarrhythmic drug and lowers blood pressure by blocking L-type calcium channels and inhibiting angiotensin-converting enzyme activity [94,95].

Barbary wolfberry fruit contains high levels of carotenoids, primarily zeaxanthin and its dipalmitate, which constitute approximately 31 to 56% of the total carotenoid content. These compounds exhibit strong antioxidant activity, protect low-density lipoprotein (LDL) from oxidative modification, and demonstrate anti-atherosclerotic effects [96,97].

Adenosine, one of the nucleoside components in glossy ganoderma, helps prevent platelet aggregation, improves microcirculation, and protects the heart by activating adenosine receptors [98,99]. Manuka honey contains methylglyoxal, found at levels of 100–1000 mg/kg, which gives it strong antibacterial properties [100] (Table 1).

## 4. Anti-Atherosclerotic Molecular Mechanisms

### 4.1. Improving Endothelial Dysfunction

**Figure 2:**
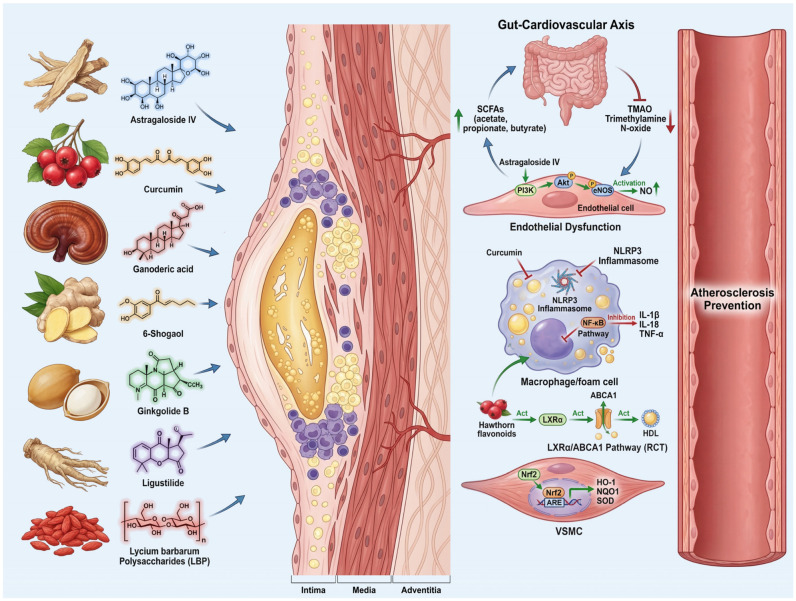
Signaling pathway network diagram of anti-atherosclerotic effects of food and medicine continuum materia medica.

In cultured endothelial cells and diabetic mouse models, astragaloside IV enhances endothelial function through activation of the PI3K/Akt/eNOS signaling pathway, leading to increased eNOS phosphorylation at serine 473 and elevated NO production. This process significantly induces vasodilation and ameliorates diabetic vascular endothelial dysfunction by inhibiting the toll-like receptor 4 (TLR4)/NF-κB signaling pathway [47,101]. However, no clinical trials have specifically evaluated the effects of astragaloside IV on endothelial function in patients with atherosclerosis. While experimental evidence from in vitro and in vivo studies indicates potential beneficial effects of astragalus on cardiomyocytes, data from high-quality clinical trials investigating the efficacy and safety of astragalus for the treatment of cardiovascular diseases are lacking [102]. Additionally, ligustilide derived from *Angelica sinensis* activates the Nrf2/HO-1 pathway, which promotes endothelial cell NO synthesis, suppresses tumor necrosis factor-alpha (TNF-α)-induced adhesion molecule expression, and reduces vascular inflammation [103] (Figure 2).

The excessive expression of endothelial cell adhesion molecules, including intercellular adhesion molecule-1 (ICAM-1), vascular cell adhesion molecule-1 (VCAM-1), and E-selectin, facilitates monocyte adhesion and transendothelial migration, which are critical events in the early stages of atherosclerosis [104]. In cell culture systems, 6-shogaol, a compound derived from ginger, reduces leukocyte–endothelial cell adhesion and transendothelial migration by inhibiting NF-κB promoter activity and suppressing lipopolysaccharide (LPS)-induced expression of ICAM-1, VCAM-1, and E-selectin [105]. Ginkgolide B inhibits ox-LDL-induced nicotinamide adenine dinucleotide phosphate (NADPH) oxidase 4 expression and reactive oxygen species (ROS) production, decreases monocyte chemoattractant protein-1 (MCP-1) and ICAM-1 expression, prevents NF-κB p65 nuclear translocation, and mitigates ox-LDL-induced endothelial dysfunction by downregulating lectin-like oxidized low-density lipoprotein receptor-1 (LOX-1) expression [106,107].

### 4.2. Regulating Lipid Metabolism

Hawthorn fruit flavonoids modulate cholesterol metabolism via multiple mechanisms. These include upregulation of low-density lipoprotein receptor (LDLR), liver X receptor alpha (LXRα), and ATP-binding cassette subfamily G member 5/member 8 expression; increased cytochrome P450 family 7 subfamily A member 1 (CYP7A1) levels to enhance bile acid synthesis; and downregulation of proprotein convertase subtilisin/kexin type 9 (PCSK9), 3-hydroxy-3-methylglutaryl-coenzyme A reductase (HMGCR), and SREBP cleavage-activating protein (SCAP) expression to suppress cholesterol synthesis. Additionally, these flavonoids activate the AMPK/SREBP-1c pathway and act as natural PPARα agonists, thereby upregulating carnitine palmitoyltransferase 1A (CPT-1A) and promoting fatty acid oxidation [27,108]. Glossy ganoderma triterpenoid compounds, such as ganoderic acid A, ganoderic acid η, and ganoderic acid K, inhibit HMG-CoA reductase activity, resulting in reduced cholesterol synthesis [109]. Furthermore, ganoderic acid A inhibits SREBP expression, which decreases intracellular cholesterol and fatty acid levels and enhances insulin sensitivity [110].

Reverse cholesterol transport (RCT) is a critical mechanism that facilitates the movement of cholesterol from peripheral tissues to the liver for metabolic processing, with ATP-binding cassette transporter A1 (ABCA1) serving as the principal mediator of cholesterol efflux [111,112]. Quercetin enhances ABCA1 expression by activating peroxisome proliferator-activated receptor gamma (PPARγ) and liver X receptor alpha (LXRα), thereby promoting cholesterol efflux from macrophages to high-density lipoprotein (HDL) and apolipoprotein A1 (apoA1) [113]. In ApoE^−^/^−^ mice, flavonoids derived from hawthorn fruit leaves significantly reduce the area of atherosclerotic lesions, increase hepatic expression of PPARα and low-density lipoprotein receptor (LDLR), inhibit foam cell formation, and promote RCT in vivo [114]. Polysaccharides from glossy ganoderma modulate lipid metabolism and enhance RCT through multiple signaling pathways, including Nrf2-Keap1, NF-κB, LXRα-ABCA1/ABCG1, CYP7A1-CYP27A1, and farnesoid X receptor-fibroblast growth factor 15 (FXR-FGF15) [115]. These preclinical findings provide a mechanistic rationale for potential lipid-lowering effects, though confirmatory human data remain limited.

### 4.3. Inhibiting Vascular Inflammation

Curcumin acts as a prototypical inhibitor of the NF-κB pathway in preclinical models. In ApoE^−^/^−^ mice, curcumin significantly reduces TLR4 expression and macrophage infiltration, and decreases aortic interleukin-1 beta (IL-1β), TNF-α, VCAM-1, and ICAM-1 expression, leading to a marked reduction in atherosclerotic lesion area [116]. Gingerols and shogaols derived from ginger inhibit IL-1β expression as well as prostaglandin E_2_ and thromboxane B_2_ production by directly inhibiting calcium-independent phospholipase A_2_ and cytosolic phospholipase A_2_ activities; among these, 10-shogaol exhibits the most pronounced effect [117]. Ginkgolide B demonstrates anti-inflammatory properties by inhibiting the PI3K/Akt pathway. In ApoE^−^/^−^ mice, it reduces plasma platelet factor 4 (PF4) and regulated upon activation, normal T cell expressed and secreted (RANTES) levels; decreases P-selectin and VCAM-1 expression in aortic plaques; and inhibits macrophage infiltration, with efficacy comparable to that of aspirin in this murine model. Direct comparison in human atherosclerosis has not been conducted [118].Curcumin specifically inhibits NLR family pyrin domain containing 3 (NLRP3) inflammasome activation by blocking potassium efflux and apoptosis-associated speck-like protein containing a CARD (ASC) oligomerization, thereby reducing IL-1β secretion. These effects are abolished in NLRP3-deficient mice, supporting NLRP3 as the primary target of curcumin in this experimental system [119]. Flavonoids from hawthorn fruit leaves reduce plasma caspase-1, NLRP3, IL-1β, IL-18, and TNF-α levels in rats fed a high-fat diet, thereby ameliorating hepatocyte steatosis and inflammatory infiltration [108].

Macrophage polarization status, specifically the balance between M1 pro-inflammatory and M2 anti-inflammatory phenotypes, significantly influences the progression of atherosclerosis [120]. Glossy ganoderma acidic polysaccharide ganoderma tsugae acidic polysaccharide-2(GTP-2) inhibits polarization toward the M1 phenotype by regulating the NF-κB signaling pathway in ApoE^−^/^−^ mice, thereby alleviating atherosclerotic lesions [121]. Ganoderic acid suppresses M1 macrophage polarization via the TLR4/MyD88/NF-κB signaling pathway, reduces the proportion of M1 macrophages within plaques, and enhances plaque stability [122].

### 4.4. Anti-Oxidative Stress

Barbary wolfberry fruit polysaccharides increase Nrf2 levels, stimulate mitochondrial biogenesis pathways, suppress MAPK pathway activation, and mitigate oxidative stress and mitochondrial toxicity caused by mixed plasticizers in human hepatoma G2 (HepG2) cells [123]. The use of Nrf2 inhibitors eliminates the protective effects of barbary wolfberry fruit polysaccharides, which supports the central role of Nrf2 in their antioxidant mechanism in this in vitro system. Additionally, barbary wolfberry fruit polysaccharides promote Nrf2 nuclear translocation, upregulate HO-1 and NQO1 expression, decrease ROS production and malondialdehyde (MDA) levels, enhance superoxide dismutase (SOD) and glutathione peroxidase (GSH-Px) activities, and preserve mitochondrial function [124]. Ginkgolide B restores sphingolipid homeostasis, lowers ceramide levels, and improves lipid metabolism and oxidative damage in hyperlipidemic rats by activating PPARα and Nrf2 pathways [125]. Curcumin reduces oxidative stress by activating the Nrf2/HO-1 axis and inhibiting NF-κB and MAPK signaling pathways, thereby exerting multi-target anti-atherosclerotic effects [126].

Nrf2 activation inhibits NF-κB activity through mechanisms such as competitive binding of the transcriptional coactivator CBP/p300, upregulation of HO-1 expression (with HO-1 metabolites CO and bilirubin exhibiting anti-inflammatory effects), and inhibition of IκB kinase activity [127]. This cross-regulatory mechanism underlies the molecular basis for the concurrent antioxidant and anti-inflammatory effects of FMC materia medica.

Phenolic compounds in honey inhibit oxidative stress by reducing ROS production, restoring antioxidant enzyme activity, and enhancing mitochondrial antioxidant status. These effects are mediated through multiple signaling pathways, including p38 MAPK, AMPK, PI3K/Akt, NF-κB, and Nrf2 [128].

### 4.5. Inhibiting Foam Cell Formation

Phenolic compounds in honey, such as quercetin, kaempferol, and apigenin, exhibit substantial anti-LDL oxidation activity. Research indicates that dark honeys, including buckwheat, honeydew, and manuka varieties, possess greater antioxidant capacity and higher total polyphenol content. These constituents are closely linked to honey’s capacity to inhibit LDL oxidation [38,129].

LOX-1 and cluster of differentiation 36 (CD36) are primary receptors involved in the uptake of oxidized LDL (ox-LDL). Ginkgolide B downregulates ox-LDL-induced LOX-1 and ICAM-1 expression and reduces cholesterol deposition in endothelial cells by inhibiting Akt phosphorylation and enhancing silent information regulator 1 (SIRT1) expression [130]. Flavonoids from hawthorn fruit downregulate LOX-1 expression by inhibiting the sPLA_2_-IIA/SCAP-SREBP2-LDLR pathway, reduce ox-LDL-induced foam cell formation in RAW264.7 macrophages, and decrease intracellular levels of total cholesterol, free cholesterol, and cholesteryl esters [131]. The translational relevance of these in vitro findings to human macrophage foam cell formation warrants further investigation.

### 4.6. Maintaining Plaque Stability

6-Shogaol inhibits vascular endothelial growth factor (VEGF)-induced endothelial cell sprouting and mouse aortic ring angiogenesis, thereby demonstrating inhibitory effects on pathological angiogenesis [105]. Astragaloside IV confers anti-fibrotic and cardiovascular protective effects via multiple mechanisms, such as regulation of collagen metabolism, inhibition of apoptosis and inflammation, antioxidative activity, and enhancement in mitochondrial function [48].

Matrix metalloproteinase (MMP)-2 and MMP-9 contribute to extracellular matrix degradation, resulting in fibrous cap thinning and increased plaque instability [132]. Ginkgolide B suppresses ox-LDL-induced MMP-1 and COX-2 expression in RAW264.7 macrophages, thereby reducing inflammatory cascades and promoting plaque stability [107].

### 4.7. Regulating the Gut Microbiota–Metabolite–Cardiovascular Axis

Glossy ganoderma spore extract has been shown to reduce serum trimethylamine N-oxide (TMAO) levels in rats with TMAO-induced cardiac dysfunction, alter gut microbiota composition by increasing the abundance of Firmicutes and Proteobacteria, and decrease the abundance of Actinobacteria and Tenericutes [133]. The spore-wall-broken polysaccharide from glossy ganoderma increases intestinal short-chain fatty acid production and G protein-coupled receptor 43 (GPR43) expression, supports intestinal barrier integrity, and reduces endotoxemia [134]. Additionally, glossy ganoderma extract modulates gut microbiota, enhances colonic butyrate production, inhibits inflammatory mediators, and regulates immune-related pathways [135].

Polysaccharides from lotus leaves, specifically RG-I-type pectin, effectively bind bile acids, inhibit cholesterol micelle solubility, and stimulate the growth of Bifidobacterium and Lactobacillus. These polysaccharides demonstrate greater efficacy in regulating glucose–lipid metabolism than flavonoids and alkaloids [66]. Flavonoid-rich extracts from lotus leaves promote brown adipose tissue thermogenesis and mitigate high-fat diet-induced obesity by modulating gut microbiota, notably increasing the abundance of Akkermansia and Alistipes [136]. Fecal microbiota transplantation experiments in mice further suggest that alterations in the microbiota induced by lotus leaves are associated with weight reduction, increased energy expenditure, and enhanced brown fat activity [66].

### 4.8. Network Regulatory Mechanisms of Multi-Component Synergy

#### 4.8.1. Component Synergy Within the Same Herb

Glossy ganoderma polysaccharides and ganoderic acid A work together to reduce inflammation. When used together, they more effectively lower NO, pro-inflammatory cytokines (IL-6, IL-1β, TNF-α), and ROS levels than when used alone. They also increase the anti-inflammatory cytokine IL-10 by targeting the TLR4/NF-κB signaling pathway [137]. Using glossy ganoderma triterpenes and polysaccharides together also helps prevent macrophages from becoming inflammatory and encourages foam cell death by affecting the neurogenic locus notch homolog protein 1 (Notch1) and delta-like ligand 4 (DLL4) pathways [138].

#### 4.8.2. Comparison with Single-Target Western Medicine Treatment

Atherosclerosis is a complex disease characterized by lipid metabolism disorders, chronic inflammation, oxidative stress, and endothelial dysfunction. Because of this, the traditional approach of using one drug for one target is often not enough. Multi-target drugs can act at multiple sites simultaneously, helping achieve more predictable results and improving patient compliance [139]. By acting on multiple disease-related targets, polypharmacology can boost treatment effectiveness, help prevent drug resistance, and lower the risk of side effects [140]. FMC materia medica may conceptually align with this approach, although empirical evidence for multi-target synergistic efficacy in human cardiovascular outcomes is currently lacking. For example, the flavonoids and triterpenoids in hawthorn fruit can act on several lipid–metabolism targets, including HMG-CoA reductase, acyl coenzyme A–cholesterol acyltransferase (ACAT), and PPARα. This multi-pathway action helps reduce the compensatory effects that can happen with single-target therapies. Similarly, polysaccharides and triterpenes from glossy ganoderma act on distinct parts of the TLR4/NF-κB pathway to enhance anti-inflammatory effects. This network-based, multi-component approach gives FMC materia medica a unique advantage in long-term cardiovascular disease prevention.

## 5. Preventive Applications: From Traditional Medicinal Cuisine to Functional Foods

### 5.1. Modernization of Traditional Medicinal Cuisine

It is difficult to conduct rigorous clinical trials for traditional medicinal cuisine. This is mainly because the formulas and their proportions differ by region and school, preparation methods are not standardized, the amount of active ingredients varies widely, and the dose–response relationships are unclear [141]. Traditional herbal trials are only about one-fifth as likely to move to full-scale studies as modern herbal trials. Major barriers include not enough feasibility assessment, poor sample-size justification, and unclear randomization methods [142]. Even though there is no strong evidence from randomized controlled trials, traditional medicinal cuisine still has important theoretical and practical value. Below are three classic medicinal cuisine formulas for cardiovascular health. The single-herb extracts from these formulas have strong support from basic and clinical research (Table 2).

### 5.2. Clinical Evidence for Standardized Extracts

Ginger and honey possess the most robust clinical support, with multiple meta-analyses demonstrating significant effects on cardiometabolic risk factors, though evidence certainty ranges from low to high depending on the outcome and honey floral source [143,144]. Curcumin, hawthorn, wolfberry, ganoderma, and ginkgo have limited RCT-level evidence: an umbrella review of 72 curcumin RCTs suggests lipid-lowering effects, yet the SPORT trial reported no significant LDL-C reduction versus placebo; hawthorn meta-analyses show functional improvement in NYHA I–III heart failure but lack hard cardiovascular endpoints; wolfberry meta-analyses (total n ≈ 259) indicate modest triglyceride reduction; a Cochrane review of ganoderma (5 RCTs, n = 398) found no significant cardiometabolic benefit; and a large ginkgo RCT (n > 3000) demonstrated no cardiovascular event reduction [102,143,144,145,146,147]. For Astragalus and Angelica sinensis, available RCTs are predominantly small-scale, Chinese-language studies of low methodological quality, while lotus leaf lacks any published clinical data [148,149].

### 5.3. Functional Food Development

Functional foods incorporate FMC bioactive components into the daily diet using advanced food engineering technologies, thereby enabling unobtrusive preventive health interventions. Enhancing bioavailability is a critical aspect of functional food development. Various pharmaceutical strategies have been established to improve the bioavailability of bioactive components, including solid dispersions, nano- and microparticles, polymeric micelles, lipid nanocarriers, and cyclodextrin complexes [150]. Studies indicate that lipid-based formulations, particularly self-emulsifying drug delivery systems (SEDDSs), can markedly increase the oral bioavailability of herbal compounds [151]. Phospholipid complex technology forms molecular complexes between bioactive components and soy phospholipids, mimicking cell membrane structures to facilitate transmembrane absorption [152]. Innovating product formats is a significant approach to modernizing the application of FMC materia medica. Ready-to-drink functional beverages utilize nanoemulsification technology to disperse lipophilic components. Probiotic–FMC combinations employ glossy ganoderma polysaccharides or barbary wolfberry fruit polysaccharides as prebiotics in conjunction with probiotics, thereby synergistically regulating intestinal microecology. Functional staple foods incorporate FMC materials into traditional diets. The study by Preciado Iñiga et al. demonstrates that traditional Mexican corn tortillas containing glossy ganoderma extract produce greater lipid-lowering effects than atorvastatin in hypercholesterolemic animal models, offering new perspectives for integrating FMC materials into traditional foods [153]. Furthermore, honey can serve as a natural sweetener to replace refined sugar, while also providing phenolic compounds and antioxidant activity [154].

A critical consideration for translating preclinical findings to functional food applications is whether the reported mechanisms are achievable at nutritionally realistic doses. Many in vitro studies employ concentrations of bioactive compounds (e.g., curcumin at 10–50 μM, quercetin at 25–100 μM) that far exceed achievable plasma concentrations after oral intake. For curcumin, oral bioavailability is <1%, with peak plasma concentrations typically below 50 nM even after high-dose supplementation (8–12 g/day), representing a >100-fold gap from concentrations used in most cell-based studies [142,155]. Similarly, flavonoid glycosides from Ginkgo biloba undergo extensive first-pass metabolism, and circulating forms (glucuronide and sulfate conjugates) may have different biological activities than the aglycones tested in vitro [156]. Polysaccharides (Astragalus, Ganoderma, Lycium barbarum) are largely non-absorbable and exert their systemic effects primarily through gut microbiota-mediated metabolite production (e.g., short-chain fatty acids) rather than direct absorption, fundamentally altering the mechanistic interpretation of their cardiovascular effects. Astragaloside IV has a reported oral bioavailability of only 2.2–3.7%, raising questions about whether the PI3K/Akt/eNOS activation observed in vitro is the primary mechanism of its in vivo cardiovascular effects. These pharmacokinetic realities underscore the need for (1) conducting mechanistic studies at physiologically achievable concentrations, (2) investigating gut microbiota-mediated biotransformation as an alternative mechanism for poorly absorbed compounds, and (3) developing bioavailability-enhanced formulations before extrapolating preclinical efficacy to clinical applications.

### 5.4. Safety Management and Drug Interactions

The safety profile of MFH substances differs substantially depending on whether they are consumed as food-level intakes (e.g., ginger as a culinary spice at 1–2 g/day, honey as a sweetener at 15–30 g/day), standardized supplement doses (e.g., EGb 761 at 120–240 mg/day, ginger capsules at 0.5–3 g/day), or concentrated extracts (e.g., high-dose curcumin at 1–8 g/day, Ganoderma lucidum extract at 1.4–3 g/day). Food-level intakes generally carry minimal risk and have centuries of safe use history. The safety concerns discussed below primarily apply to standardized supplements and concentrated extracts at pharmacological doses.

Although FMC materia medica are derived from foods, the pharmacological activities of high-dose extracts and their interactions with Western medicines warrant careful consideration (Table 3). The American Heart Association scientific statement indicates that the use of complementary and alternative medicine products is increasingly common among heart failure patients, yet many products lack sufficient safety and efficacy data. Developing a risk management framework grounded in the “food–drug” continuum is crucial for the safe application of FMC products [157]. Ginkgo seed contains ginkgotoxin; excessive consumption can lead to tonic–clonic seizures, nausea, vomiting, and symptoms of neurotoxicity, with children being particularly susceptible [158,159,160]. Ginkgo seed also exhibits anti-PAF effects. Clinical case reports have documented bleeding events when *Ginkgo biloba* is combined with anticoagulant drugs [102], although controlled studies indicate that it does not significantly alter hemostatic function [161]. However, the large GEM study (n = 3069, 6.1-year follow-up) observed a numerically higher rate of hemorrhagic stroke in the ginkgo group (16 vs. 8 events), though this difference was not statistically significant [162]. As a precaution, patients receiving anticoagulant or antiplatelet therapy should consult their physicians before using ginkgo seed products, and those undergoing elective surgery should discontinue use 7 days prior to the procedure. High-dose ginger (>4 g/day) may exert antiplatelet effects; therefore, high-dose supplements should be discontinued 3 days before surgery during the perioperative period [163]. High-dose curcumin may inhibit platelet aggregation and suppress CYP3A4 and CYP2C9 enzyme activities; monitoring is recommended when curcumin is combined with statins, warfarin, or similar agents [164,165].

Glossy ganoderma exhibits immunomodulatory properties; patients receiving immunosuppressants after organ transplantation should exercise caution and discontinue immunosuppressants 1 week before and after surgery. Barbary wolfberry fruit exhibits immune-activating effects; patients with active autoimmune diseases should use caution, as case reports have documented elevated INR when barbary wolfberry fruit is combined with warfarin. Hawthorn fruit has positive inotropic and vasodilatory effects; in heart failure patients with left ventricular ejection fraction (LVEF) ≤ 35%, it may increase the risk of disease progression. Monitoring of digoxin blood concentrations is required when hawthorn fruit is used concurrently with digoxin [157,166] (Table 4).

## 6. Summary and Outlook

This review systematically evaluates research progress on ten FMC materia medica—hawthorn fruit, ginkgo seed, milkvetch root, turmeric, ginger, glossy ganoderma, Angelica sinensis, barbary wolfberry fruit, lotus leaf, and honey—in the prevention and treatment of AS. These materia medica demonstrate cardiovascular protective effects via a multi-component, multi-target, and multi-pathway network regulatory mechanism. Notably, significant advancements have been made in identifying bioactive components and elucidating molecular mechanisms. Meta-analyses have provided clinical evidence supporting the beneficial effects of ginger and honey on cardiovascular metabolic risk factors. Despite these advances, several challenges persist. Scientifically, while most materia medica exhibit effects at the biological mechanism level, current clinical studies are limited by small sample sizes and a lack of large-scale randomized controlled trials (RCTs) with major adverse cardiovascular events as endpoints. Consequently, existing data are insufficient to support clinical practice recommendations [102]. Industrially, standardizing herbal products remains difficult due to the complex, often incompletely characterized mixtures found in materia medica. Substantial variation in component composition between manufacturers and batches, coupled with inadequate standardization and quality control, poses significant concerns [155]. From a regulatory perspective, global frameworks for dietary supplements and herbal products remain fragmented, with inconsistent approval requirements across countries, thereby impeding international trade and diminishing consumer confidence [167,168].

This review references network pharmacology and molecular docking as tools for identifying bioactive components and elucidating mechanisms. However, these computational methods have inherent limitations that must be acknowledged. Molecular docking predictions are constrained by scoring function inaccuracies, with many compounds showing high docking scores but failing in preclinical validation [169,170]. Docking results cannot distinguish between agonist and antagonist activity, and the selection of correct binding sites and poses remains challenging [169]. Network pharmacology, while valuable for generating hypotheses about multi-target interactions, relies heavily on existing database annotations that may be incomplete or biased toward well-studied targets [171,172]. Predicted drug–target interactions require experimental validation, as network-based predictions reflect statistical associations rather than confirmed biological activities [172]. Furthermore, the “multi-component–multi-target–multi-pathway” paradigm, while conceptually appealing, can lead to overinterpretation when network predictions are presented without experimental confirmation. The complexity of biological systems means that network models inevitably simplify the actual pharmacological landscape, and predicted synergistic effects may not materialize in vivo due to pharmacokinetic constraints, tissue distribution differences, and dose-dependent effects [173]. Future studies should integrate computational predictions with rigorous experimental validation at physiologically relevant concentrations.

Future research should prioritize integrating metagenomics and metabolomics technologies to develop precision nutrition intervention models based on gut microbiota. The concept of “gut microbiota availability,” as proposed by Chen et al., offers a novel explanatory framework for understanding the efficacy of materia medica with low bioavailability but high bioactivity [174]. Additionally, promoting international regulatory harmonization is essential to establishing risk-stratified approval frameworks. This approach would streamline approval processes for materials with established safety profiles from traditional use, while ensuring rigorous evaluation for innovative formulations and high-dose extracts. The FMC concept reflects a strong alignment between the traditional Chinese medicine (TCM) philosophy of “preventive treatment of disease” and the modern preventive medicine principle of “food as medicine.” By advancing scientific evidence, improving quality control, and enhancing regulatory coordination, FMC materia medica are poised to play an increasingly significant role in atherosclerosis prevention and health promotion. These efforts will contribute to the development of nutritional intervention strategies with distinct Chinese characteristics for the prevention and management of chronic diseases.

## Figures and Tables

**Table 1:** Major bioactive components of ten food and medicine continuum materia medica.

Herb	Major Bioactive Components	Main Chemical Structure	Primary Cardiovascular Activities
Hawthorn fruit	Hyperoside, ursolic acid	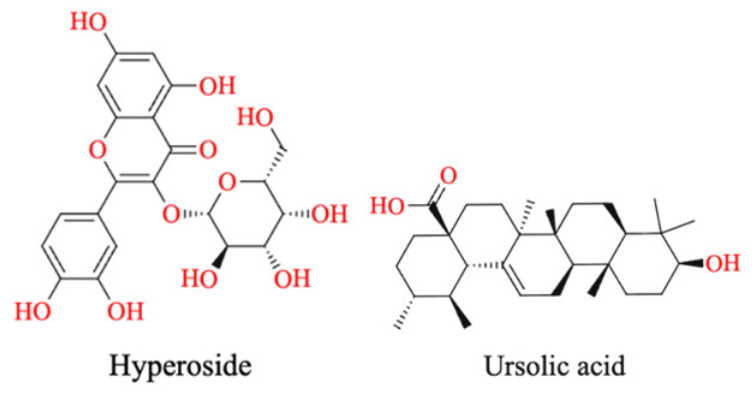	Antioxidant, lipid regulation, inhibits LDL oxidation, inhibits HMG-CoA reductase, activates Nrf2
Ginkgo seed	Ginkgetin, ginkgolide B	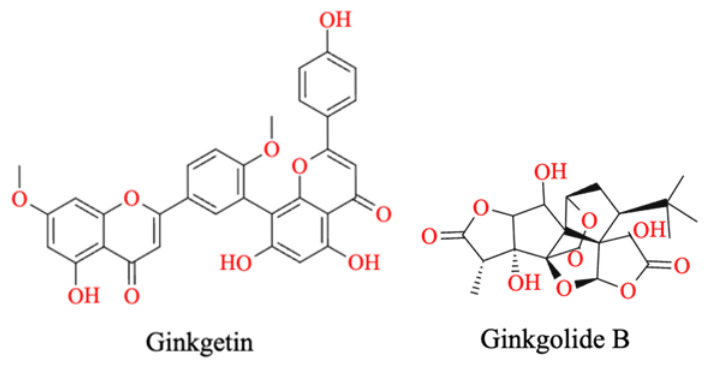	Antioxidant, anti-inflammatory, anti-PAF, neuroprotection
Milkvetch root	Astragaloside IV	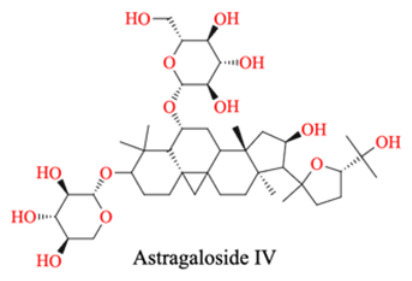	Improves endothelial function, anti-fibrosis
Turmeric	Curcumin	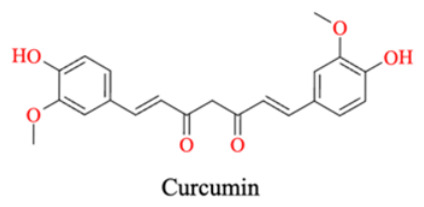	Anti-inflammatory, antioxidant
Ginger	6-Gingerol	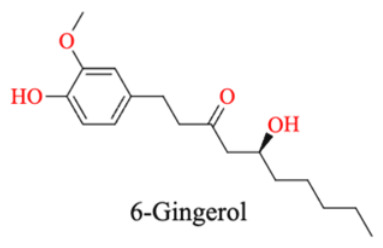	Antioxidant, anti-inflammatory, antiplatelet
Glossy ganoderma	Ganoderic acid A	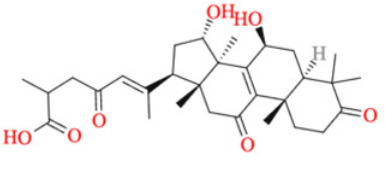	Lipid regulation, anti-inflammatory
Angelica sinensis	Ferulic acid, ligustilide	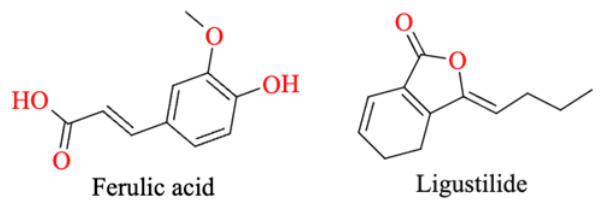	Antithrombotic, vasodilation, antiplatelet
Barbary wolfberry fruit	Zeaxanthin	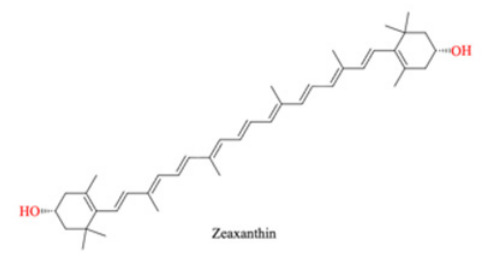	Antioxidant, anti-LDL oxidation
Lotus leaf	Nuciferine	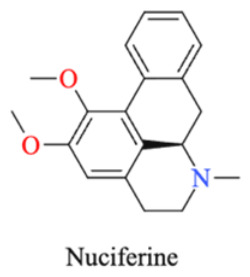	Lipid regulation and weight loss, antiarrhythmic
Honey	Quercetin	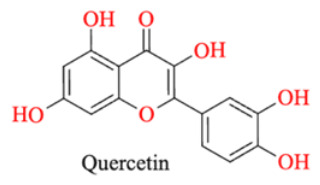	Antioxidant, anti-LDL oxidation

**Table 2:** Three classic medicinal cuisine formulas.

Medicinal Cuisine Name	Competition and Preparation	Traditional Functions	Target Population	Modern Mechanistic Research
Hawthorn fruit–lotus leaf lipid-regulating tea	Dried hawthorn fruit 10 g, dried lotus leaf 6 g; steep in boiling water or decoct for 15 min	Promotes digestion and resolves accumulation, raises the clear and lowers the turbid	Hyperlipidemia, pre-obesity	Hawthorn fruit flavonoids inhibit cholesterol synthesis; nuciferine activates AMPK
Milkvetch root–Angelica sinensis stewed chicken	Milkvetch root 30 g, Angelica sinensis 10 g, chicken 500 g; stew for 1.5 h	Tonifies qi and nourishes the blood, invigorates the blood and unblocks collaterals	Qi-blood deficiency type, postoperative recovery	Astragaloside IV improves endothelial function; ferulic acid is antithrombotic
Glossy ganoderma–Poria spirit-calming congee	Glossy ganoderma powder 3 g, Poria 15 g, polished rice 100 g; cook as congee	Calms the heart and spirit, strengthens the spleen and promotes diuresis	Insomnia, anxiety, low immunity	Glossy ganoderma polysaccharides provide immunomodulation; triterpenes are anti-inflammatory

**Table 3:** Summary evidence matrix: anti-atherosclerotic evidence for ten FMC materia medica.

FMC Substance	Major Bioactive Compounds	Lipid Regulation	Anti- Inflammation	Endothelial Protection	Anti- Oxidant	Gut Microbiota Modulation	Highest Level of Clinical Evidence
Hawthorn fruit	Hyperoside, ursolic acid, procyanidin B2	++ (animal)	+ (in vitro)	+ (animal)	++ (in vitro/animal)	–	Meta-analyses of RCTs (heart failure symptoms; no AS endpoints)
Ginkgo seed	Ginkgolides, bilobalide, biflavonoids	+ (in vitro)	++ (animal)	++ (in vitro/animal)	++ (in vitro/animal)	–	No AS-specific RCTs
Milkvetch root	Astragaloside IV, calycosin	+ (animal)	+ (in vitro/animal)	+++ (in vitro/animal)	+ (in vitro)	–	Small observational studies only (Chinese language)
Turmeric	Curcumin	+ (animal)	+++ (in vitro/animal)	+ (in vitro)	+++ (in vitro/animal)	+ (animal)	Limited RCTs (biomarkers only; no AS endpoints)
Ginger	6-Gingerol, 6-shogaol	+ (animal)	++ (in vitro/animal)	+ (in vitro)	++ (in vitro/animal)	–	Meta-analyses supporting BP, TG, LDL-C reduction
Glossy ganoderma	Ganoderic acid A, β-glucans	++ (animal)	++ (in vitro/animal)	–	+ (in vitro)	++ (animal)	Cochrane review: no effect on CV risk factors (3 RCTs in T2DM)
Angelica sinensis	Ferulic acid, ligustilide	–	+ (in vitro)	++ (in vitro/animal)	+ (in vitro)	–	No AS-specific clinical trials
Barbary wolfberry fruit	Zeaxanthin, LBP	–	+ (in vitro)	–	++ (in vitro/animal)	–	No AS-specific clinical trials
Lotus leaf	Nuciferine, RG-I pectin	++ (animal)	–	–	–	++ (animal, FMT confirmed)	No AS-specific clinical trials
Honey	Quercetin, phenolic acids	+ (clinical)	+ (in vitro)	–	++ (in vitro/clinical)	–	Meta-analysis (conflicting results on lipids)

Table notes: Evidence strength: +++ = multiple consistent preclinical studies with strong mechanistic support; ++ = moderate preclinical evidence; + = preliminary or limited evidence; – = no or negligible evidence. The evidence source (in vitro, animal, clinical) is indicated in parentheses. “AS endpoints” refers to atherosclerotic plaque burden, carotid intima-media thickness, or major adverse cardiovascular events. FMT = fecal microbiota transplantation; LBP = Lycium barbarum polysaccharides; T2DM = type 2 diabetes mellitus; BP = blood pressure; TG = triglycerides.

**Table 4:** Safety warnings and drug interactions.

Herb	Risk Mechanism	Specific Manifestations	High-Risk Populations/Drugs
Ginkgo semen	Ginkgotoxin, anti-PAF, antiplatelet aggregation	Tonic–clonic seizure, prolonged bleeding time	Patients with epilepsy; heavy consumers; warfarin, aspirin, clopidogrel users
Ginger	High dose (>4 g/day) inhibits platelets	Increased bleeding risk	Perioperative period, anticoagulation therapy patients
Glossy ganoderma	Immunomodulatory effects	Adverse event risk increased 1.67-fold (non-serious)	Post-organ transplant (immunosuppressant users); active autoimmune disease
Hawthorn fruit	Positive inotropic effect, vasodilation	May enhance vasodilator and digoxin effects	Heart failure patients with LVEF ≤ 35%; digoxin users
Turmeric	High dose inhibits platelet aggregation; inhibits CYP3A4/2C9	Theoretically increases bleeding risk; affects drug metabolism	Anticoagulation therapy patients, perioperative period; statin users
Barbary wolfberry fruit	Immune activation; may enhance warfarin effects	Active autoimmune disease risk; elevated INR	Active systemic lupus erythematosus, rheumatoid arthritis; warfarin users

## Data Availability

The original contributions presented in this study are included in the article. Further inquiries can be directed to the corresponding author.

## Abbreviations

The following abbreviations are used in this manuscript:

CVD	Cardiovascular disease
AS	Atherosclerosis
FMC	Food and medicine continuum
TCM	Traditional Chinese medicine
AMPK	Adenosine 5′-monophosphate-activated protein kinase
SREBP-1c	Sterol regulatory element-binding protein-1c
PPARα	Peroxisome proliferator-activated receptor alpha
PAF	Platelet-activating factor
Nrf2	Nuclear factor erythroid 2-related factor 2
PI3K	Phosphatidylinositol 3-kinase
Akt	Protein kinase B
eNOS	Endothelial nitric oxide synthase
HMG-CoA	3-hydroxy-3-methylglutaryl-coenzyme A
NF-κB	Nuclear factor kappa-light-chain-enhancer of activated B cells
MAPK	Mitogen-activated protein kinase
HO-1	Heme oxygenase-1
NQO1	NAD(P)H quinone oxidoreductase 1
RG-I	Rhamnogalacturonan-I
COX	Cyclooxygenase
TXA2	Thromboxane A2
NO	Nitric oxide
iNOS	Inducible nitric oxide synthase
ARE	Antioxidant response element
LDL	Low-density lipoprotein
TLR4	Toll-like receptor 4
TNF-α	Tumor necrosis factor-alpha
ICAM-1	Intercellular adhesion molecule-1
VCAM-1	Vascular cell adhesion molecule-1
NADPH	Nicotinamide adenine dinucleotide phosphate
ROS	Reactive oxygen species
LOX-1	Lectin-like oxidized low-density lipoprotein receptor-1
LDLR	Low-density lipoprotein receptor
LXRα	Liver X receptor alpha
CYP7A1	Cytochrome P450 family 7 subfamily A member 1
SCAP	SREBP cleavage-activating protein
RCT	Reverse cholesterol transport
ABCA1	ATP-binding cassette transporter A1
HDL	High-density lipoprotein
IL-1β	Interleukin-1 beta
NLRP3	NLR family pyrin domain containing 3
MMP	Matrix metalloproteinase
TMAO	Trimethylamine N-oxide
